# Health and wellbeing of staff working at higher education institutions globally during the post-COVID-19 pandemic period: evidence from a cross-sectional study

**DOI:** 10.1186/s12889-024-19365-1

**Published:** 2024-07-11

**Authors:** Muhammad Aziz Rahman, Pritimoy Das, Louisa Lam, Sheikh M. Alif, Farhana Sultana, Masudus Salehin, Biswajit Banik, Bindu Joseph, Parul Parul, Andrew Lewis, Dixie Statham, Joanne Porter, Kim Foster, Sheikh Mohammed Shariful Islam, Wendy Cross, Alycia Jacob, Susan Hua, Qun Wang, Sek Ying Chair, Wai Tong Chien, Sri Widati, Ira Nurmala, Ni Nyoman Tri Puspaningsih, Majeda Hammoud, Khatijah Omar, Muhammad Abi Sofian Abdul Halim, Mohammed Gamal-Eltrabily, Georgina Ortiz, Turkiya Saleh Al Maskari, Salwa Saleh Mohammed Al Alawi, Badriya Saleh Al-Rahbi, Judie Arulappan, Akhlaq Ahmad, Nahed Al Laham, Ilias Mahmud, Ibrahim Alasqah, Habib Noorbhai, Shao-Liang Chang, Yi-Lung Chen, Mehmet Fatih Comlekci, Oguz Basol, Basema Saddik, Rick Hayman, Remco Polman

**Affiliations:** 1 Institute of Health and Wellbeing, Federation University Australia, Berwick, VIC 3806 Australia; 2 Collaborative Evaluation and Research Centre (CERC), Federation University Australia, Churchill, Australia; 3 School of Nursing, Midwifery and Paramedicine, Australian Catholic University, Melbourne, Australia; 4 School of Health Sciences, The University of Melbourne, Melbourne, Australia; 5 Telstra Health, Melbourne, Australia; 6 School of Nursing, Institute of Health & Management, North Melbourne, VIC 3051 Australia; 7 Institute for Physical Activity and Nutrition, Deakin University, Burwood, Australia; 8 School of Nursing, Shenzhen University, Shenzhen, China; 9 The Nethersole School of Nursing, The Chinese University of Hong Kong, Shatin, Hong Kong, China; 10 Universitas Airlangga, Surabaya, Indonesia; 11 Faculty of Medicine, Kuwait University, Kuwait City, Kuwait; 12 Institute of Tropical Biodiversity & Sustainable Development, Universiti Malaysia Terengganu, Terengganu, Malaysia; 13 División de Ciencias de La Salud, Universidad Anáhuac Querétaro, Querétaro, México; 14 Oman College of Health Sciences-South Sharquiya, South Sharquiya, Oman; 15 Sultan Qaboos University, AlKhoudh, Oman; 16 University of the Punjab, Lahore, Pakistan; 17 Department of Laboratory Medicine, Al Azhar University-Gaza, Gaza Strip, Palestine; 18 School of Health, University of New England, Armidale, Australia; 19 BRAC James P. Grant School of Public Health, BRAC University, Dhaka, Bangladesh; 20 Department of Community, Psychiatric and Mental Health Nursing, College of Nursing, Qassim University, Buraydah, Saudi Arabia; 21 BEAHT Research Centre, University of Johannesburg, Johannesburg, South Africa; 22 Asia University, Wufeng, Taiwan; 23 Kirklareli University, Kirklareli, Turkey; 24 College of Medicine, University of Sharjah, Sharjah, United Arab Emirates; 25 School of Population Health, University of New South Wales, Kennington, Australia; 26 Northumbria University, Newcastle-Upon-Tyne, UK

**Keywords:** Health, Job insecurity, Resilient coping, University staff, Mental health

## Abstract

**Background:**

The ongoing global crisis of Higher Education (HE) institutions during the post-COVID-19 pandemic period has increased the likelihood of enduring psychological stressors for staff. This study aimed to identify factors associated with job insecurity, burnout, psychological distress and coping amongst staff working at HE institutions globally.

**Methods:**

An anonymous cross-sectional study was conducted in 2023 with staff at HE institutions across 16 countries. Job insecurity was measured using the Job Insecurity Scale (JIS), burnout using the Perceived Burnout measure question, psychological distress using the Kessler Psychological Distress Scale (K10), and coping using the Brief Resilient Coping Scale. Multivariable logistic regression with a stepwise variable selection method was used to identify associations.

**Results:**

A total of 2,353 staff participated; the mean age (± SD) was 43(± 10) years and 61% were females. Most staff (85%) did not feel job insecurity, one-third (29%) perceived burnout in their jobs, more than two-thirds (73%) experienced moderate to very high levels of psychological distress, and more than half (58%) exhibited medium to high resilient coping. Perceived job insecurity was associated with staff working part-time [Adjusted Odds Ratio 1.53 (95% Confidence Intervals 1.15–2.02)], having an academic appointment [2.45 (1.78–3.27)], having multiple co-morbidities [1.86 (1.41–2.48)], perceived burnout [1.99 (1.54–2.56)] and moderate to very high level of psychological distress [1.68 (1.18–2.39)]. Perceived burnout was associated with being female [1.35 (1.12–1.63)], having multiple co-morbidities [1.53 (1.20–1.97)], perceived job insecurity [1.99 (1.55–2.57)], and moderate to very high levels of psychological distress [3.23 (2.42–4.30)]. Staff with multiple co-morbidities [1.46 (1.11–1.92)], mental health issues [2.73 (1.79–4.15)], perceived job insecurity [1.61 (1.13–2.30)], and perceived burnout [3.22 (2.41–4.31)] were associated with moderate to very high levels of psychological distress. Staff who perceived their mental health as good to excellent [3.36 (2.69–4.19)] were more likely to have medium to high resilient coping.

**Conclusions:**

Factors identified in this study should be considered in reviewing and updating current support strategies for staff at HE institutions across all countries to reduce stress and burnout and improve wellbeing.

**Supplementary Information:**

The online version contains supplementary material available at 10.1186/s12889-024-19365-1.

## Background

During the pandemic, government restrictions greatly affected higher education (HE) institutions, altering teaching delivery and learning modalities. Face-to-face classes were cancelled, and delivery shifted to online with tight deadlines and limited support. These abrupt changes were particularly challenging for staff and students in practical and lab-oriented courses [1]. The work-related demands combined with job insecurity stemming from significant financial losses in HE institutions globally, affected the wellbeing of staff [2]. HE staff, like others, faced public health mandates, including social isolation, working from home, managing home-schooling children or caring for relatives with COVID-19 [3]. These factors compounded work-related stress.

Staff working at HE institutions had always been exposed to varied sources and types of work-related stresses impacting on their health and wellbeing. During the post-pandemic era, HE institutions and staff faced further challenges globally. The most recent Australian National Tertiary Education Union’s four-year survey data (2020–23) with over 6,200 staff showed the reporting of poor work environments, poor psychosocial safety, high levels of burnout, extreme tiredness, anxiety, or depression [4]. In addition, HE institutions of many developed countries like Australia and UK had significant financial impact, primarily due to loss of international students, which they tried to recover by adopting a number of cost-saving strategies such as organisational restructure, reducing staff costs including redundancies, early retirements, terminations, and reducing non-salary expenditures following the pandemic period [5]. Such changes also impacted on the remaining staff, who got increased workload, complexities in their role impacting on their heath and wellbeing.

The impact of COVID-19 on HE staff varied globally due to diverse political, cultural, environmental, and geographical factors, and differing national responses to the pandemic. Numerous studies have explored the impact of COVID-19 on HE students [6], however, very few have examined the health and wellbeing of staff who managed and supported students directly. Within the environment of financial losses with resultant cost-recovery approaches at HE institutions in the post-pandemic period, staff are concerned of their own job security besides adopting with increased work demand and performance expectations leading to work-stress [5]. Prior evidence indicates that work-related stress derives from teaching stress, research stress and administrative stress leading to emotional burnout [7]. Being resilient is one of the key strategies to overcome negative impacts on mental health, which has been shown effective amongst students during the pandemic adopting new learning environments [8]. Therefore, it’s also important to examine the coping strategies of staff dealing with the changed environment at HE institutions. No evidence-based evaluations of psychological factors affecting HE staff in various countries exist. Therefore, this multi-country study aimed to assess the psychological wellbeing and profession-related stress of staff across diverse global HE institutions.

We aimed to assess job insecurity, burnout, psychological distress, and coping among staff at HE institutions in 16 countries during the post-pandemic period. This exploratory study may inform post-pandemic health and wellbeing, and guide future interventions and policies to enhance wellbeing and reduce stress and burnout for this population.

## Methods

### Study design and settings

A cross-sectional study involving 16 countries was conducted from May to August 2023 using Qualtrics (Qualtrics^XM^ is a licensed data collection software). The countries included Australia, Mainland China, Hong Kong (China), Indonesia, Kuwait, Malaysia, Oman, Pakistan, Palestine, Saudi Arabia, South Africa, Taiwan (China), Turkey, the United Arab Emirates (UAE), Mexico, and the United Kingdom (UK).

### Study population

#### Inclusion criteria

All staff from 18 higher education institutions from those 16 countries, regardless of employment status or role, were invited to participate. Countries and institutions were selected based on the existing research collaboration of the first author (MAR).

#### Exclusion criteria

Participants who completed the survey in under 1 min or over 60 min were excluded to prevent information bias.

### Sampling

Population and stress varied across countries. To ensure adequate statistical power, a minimum sample size of 385 was established for each participating organisation, despite variations in population size and stress prevalence.

### Data collection

An online-based questionnaire was developed using a licensed Qualtrics platform hosted by Federation University Australia. Ethics approval was obtained from Federation University Australia, the Australian Catholic University, and corresponding organisations in each participating country. (See the appendix for details).

Given the global multicultural nature of participation, participants could select their preferred language on the first screen. The languages were Arabic, Bhasa Indonesia, Chinese (Traditional and Modern), English, Spanish and Turkish. The Plain Language Information Statement (PLIS), consent form and survey were translated and back-translated from the English version. Lead investigators conducted pilot testing in UAE (Arabic), Indonesia (Bahasa Indonesia), China, Hong Kong and Taiwan (Traditional and Modern Chinese), Mexico (Spanish), and Turkey (Turkish). The second screen of the survey included the PLIS and consent form, affirming anonymity and voluntary participation. Only consenting participants could proceed to the next screen to access the survey questions.

Email invitations containing the online survey link were distributed to all staff in the participating organisations through the assistance of the lead investigator from each organisation. The first reminder email was sent three weeks after the initial email, followed by a second reminder three weeks later.

### Study tool

A structured survey based on a previous global study led by the first author (MAR) was used [9]. Participants completed a 31-item survey consisting of items on countries/regions, socio-demographic factors (age, gender, highest level of education, work experience duration, living arrangement), profession details (job and appointment type, level, employment status), self-reported health conditions, and behavioural risk factors (smoking, alcohol consumption, physical activity), COVID-19 related experiences (positive tests, long-COVID symptoms, vaccination status), work-related concerns (job insecurity, burnout), psychological impact (mental health perception, distress), coping strategies, and physical health indicators (blood pressure, lipid profile, blood sugar, healthcare visits).

Variables were measured using validated and reliable tools: (1) job insecurity, with the 4-item Job Insecurity Scale (JIS) [10], (2) burnout with a single-item perceived burnout question [11], (3) psychological distress with the 10-item Kessler Psychological Distress Scale (K-10) [12, 13], and (4) coping with the 4-item Brief Resilient Coping Scale (BRCS) [14]. The estimated completion time for the survey was 20 min.

### Data analyses

Data were analysed using STATA v.18. Continuous variables (age, years of experience, total scores for each outcome measure were presented as mean and standard deviations. Categorical variables underwent further inferential analyses. The JIS total scores were dichotomised into no job insecurity (1–3) and perceived job insecurity (4–5) [10] [6]. Perceived burnout scores categorised as no burnout (1–4) and perceived burnout (5–7) [11] [7]. Psychological distress scores were categorised as low (10–15) and moderate to very high (16–50) [12, 13] [8, 9]. Coping was categorised as low (4–13) and medium to high (14–20) based on the BRCS scoring [14–16].

Univariate analyses determined associations between outcomes and factor variables, yielding odds ratios (ORs) and 95% confidence intervals (CIs).. Multivariate logistic regression along with stepwise variable selection analysis was performed to investigate the controlled effect of factors such as age, sex, highest academic qualification, smoking, alcohol intake, country of residence, type and level of appointment, employment status and comorbid conditions on job insecurity, perceived burnout, psychological distress and resilient coping. Variables with a cut off value of *p* < 0.05 were identified from the initial chi-squared tests, which were included as potential confounders in the multivariate analyses. Similar approaches have been widely supported as a methodological concept in statistical analysis [17]. For country-wise comparisons, the reference country was selected based on the lowest prevalence of perceived job insecurity, perceived burnout, moderate to very high psychological distress, and medium to high resilience coping. Other countries and areas were organised chronologically by prevalence (lowest to highest) for each outcome before conducting the multivariate analyses. To deal with missing values, actual responses received for each variable were considered and reported accordingly as proportions in the descriptive analyses; actual responses were used for inferential analyses. We also performed a sensitivity analysis excluding the missing values and a multiple imputation technique was also conducted. We did not observe any changes in the effect estimates.

To avoid potential type I errors arising from multiple comparisons, significance threshold can be minimised by adjusting the *p*-values [18]. Given the number of four dependent/outcome variables, significance threshold for multivariate analyses were adjusted accordingly (0.05/4 = 0.0125). Therefore, the cut-off of *p* < 0.01 was used as statistical significance for all findings of the multivariate analyses in this study.

## Results

A total of 22,597 staff across 16 countries were invited via emails to participate, 2,353 (10%) responded. The response rate varied across countries, ranging from 70% (155 responses out of 220 staff) in Malaysia to 1% (47 responses out of 6,900 staff) in Saudi Arabia. (Table S.1) Details of socio-demographic data and characteristics of study population are presented in Table 1. The proportion of missing values did not exceed over 3% for our study variables. Most staff (*n* = 2,004, 85%) did not report job insecurity. However, 29% (*n* = 684) reported experiencing burnout. Twenty-three percent (*n* = 527) perceived their own mental health to be fair to poor. More than two-thirds of participants (*n* = 1,685, 73%) experienced moderate to very high levels of psychological distress, and 58% (*n* = 1,317) exhibited medium to high resilient coping. (Tables S.2, S.3, S.4).

**Table 1:** Characteristics of the study population

Characteristics	Total, n(%)
Total study participants	2353(100)
Country/area of residence	2353
Australia	424(18.0)
Mainland China	137(5.8)
Hong Kong, China	186(7.9)
Indonesia	61(2.6)
Kuwait	96(4.1)
Malaysia	155(6.6)
Mexico	316(13.4)
Oman	86(3.7)
Pakistan	37(1.6)
Palestine	91(3.9)
Saudi Arabia	47(2.0)
South Africa	65(2.8)
Taiwan, China	46(2.0)
Turkey	147(6.2)
United Arab Emirates	115(4.9)
United Kingdom	344(14.6)
Age (in years)	2306
Mean (± SD)	42.9 (± 10.4)
Age groups	2306
18–29 years	227(9.8)
30–59 years	1915(83.0)
≥ 60 years	164(7.1)
Gender	2348
Male	877(37.4)
Female	1436(61.2)
Other	10(0.4)
Prefer not to say	25(1.1)
Highest educational/vocational qualification	2335
Certificate/Diploma/Trade qualifications	209(9.0)
Bachelors level	532(22.8)
Masters level	756(32.4)
Doctoral level	838(35.9)
Duration of work in higher education institutions (in years)	2299
Mean (± SD)	12.2 (± 8.8)
Duration of work	2299
< 5 years	514(22.4)
5 to < 10 years	496(21.6)
≥ 10 years	1289(56.1)
Current living status	2345
Live without family members (on your own/shared house)	380(16.2)
Live with family members (spouse/partners/siblings/children)	1943(82.9)
Others	22(0.9)
Types of job (multiple responses)	2353
Teaching	1344 (57.1)
Research	913 (38.8)
Admin	1260 (53.5)
Leadership	482 (20.5)
Types of appointment	2347
Professional/Admin	908(38.7)
Academic (Teaching and/or Research)	1324(56.4)
Leadership	115(4.9)
Levels of appointment	2334
Lecturer	348(14.9)
Senior Lecturer/Assistant Professor	451(19.3)
Associate Professor	202(8.7)
Professor	240(10.3)
Others	1030(44.1)
Prefer not to say	63(2.7)
Current employment condition	2353
Full time	1925(81.8)
Part time	428(18.2)
Self-reported co-morbidities	2353
No	1254 (53.3)
Single co-morbidity	580 (24.6)
Multiple co-morbidities	519 (22.1)
Self-reported co-morbidities	2353
No	1254 (53.3)
Mental health issue	293 (12.5)
Other co-morbidity	806 (34.3)
Smoking	2353
Never smoker	1793 (76.2)
Ever smoker (Current and Ex)	560 (23.8)
Increased smoking over the last 6 months (current smokers)	253
Yes	88 (34.8)
No	165 (65.2)
Current alcohol drinking	2352
Yes	937 (39.8)
No	1415 (60.2)
Frequency of alcohol drinking in the last 6 months	937
Everyday	23 (2.5)
More than 5 times a week	48 (5.1)
2–4 times a week	225 (24.0)
Once a week	148 (15.8)
Only on weekends	168 (17.9)
On special occasions	325 (34.7)
Increased alcohol drinking over the last 6 months	935
Yes	138 (14.8)
No	797 (85.2)
Physical activity for at least 30-min in the past week (days)	2349
Mean (± SD)	3.7 (± 2.2)
Physical activity for at least 30-min in the past week (days)	2349
None	489 (20.8)
1–3 days	1124 (47.9)
4–7 days	736 (31.3)
Experience related to COVID-19 pandemic	2350
No known exposure to COVID-19	791 (33.7)
Tested positive for COVID-19	1559 (66.3)
Frequency of positive tests for COVID-19	2177
Mean (± SD)	1 (± 1.1)
Symptoms of long COVID	2270
No	1608 (70.8)
Yes	662 (29.2)
Doses of COVID vaccine taken	2347
None	56 (2.4)
Double	635 (27.1)
Triple or more	1656 (70.6)
Last checked/measured blood pressure	2259
Never checked blood pressure	174 (7.7)
Checked within last 6 months	1430 (63.3)
Checked > 6 months ago	674 (29.8)
Last checked/measured blood lipid profile	2254
Never checked blood lipid profile	659 (29.2)
Checked within last 6 months	803 (35.6)
Checked > 6 months ago	792 (35.1)
Last checked/measured blood sugar	2258
Never checked blood sugar	479 (21.2)
Checked within last 6 months	970 (43.0)
Checked > 6 months ago	809 (35.8)
Last visited a healthcare provider for general health assessment	2263
Never visited for general health assessment	382 (16.9)
Visited within last 6 months	1003 (44.3)
Visited > 6 months ago	878 (38.8)

### Job insecurity

Univariate analyses showed associations between perceived job insecurity and several variables (Table 2). After adjusting for potential confounders, perceived job insecurity was associated with: academic appointments (teaching and/or research), part-time employment, multiple co-morbidities, perceived burnout, and moderate to very high levels of psychological distress. Staff having recent blood lipid profile checks and perceiving their mental health as good to excellent did not report job insecurity in their current positions.

**Table 2:** Predictors for job insecurity among the study participants (based on the JIS scale)

Characteristics	No job insecurity (total score 1–3)		Job insecurity (total score 4–5)		Unadjusted analyses			Adjusted analyses#		
	n	%	n	%	*p*	ORs	95% CIs	*p*	AORs	95% CIs
Total study participants	2004		349							
Age groups										
18–29 years	188	82.82	39	17.18		Ref		Not selected in the multivariate model		
30–59 years	1638	85.54	277	14.46	0.276	0.81	0.56–1.17			
≥ 60 years	138	84.15	26	15.85	0.728	0.91	0.53–1.56			
Gender										
Male	756	86.2	121	13.8		Ref		Not selected in the multivariate model		
Female	1219	84.89	217	15.11	0.385	1.11	0.87–1.41			
Highest educational/vocational qualification										
Certificate/Diploma/Trade qualifications	182	87.1	27	12.92		Ref		Not selected in the multivariate model		
Bachelors level	455	85.53	77	14.47	0.584	1.14	0.71–1.82			
Masters level	630	83.33	126	16.67	0.190	1.34	0.86–2.10			
Doctoral level	721	860.04	117	13.96	0.695	1.1	0.69–1.71			
Duration of work										
< 5 years	422	82.1	92	17.9		Ref			Ref	
6–10 years	414	83.47	82	16.53	0.565	0.91	0.65–1.26	Not selected in the multivariate model		
> 10 years	1120	86.89	169	13.11	***0.009***	***0.69***	***0.52–0.91***	0.032	0.77	0.61–0.98
Current living status										
Live without family members (on your own/shared house)	305	80.26	75	19.74		Ref			Ref	
Live with family members (spouse/partners/siblings/children)	1671	86	272	14	***0.004***	***0.66***	***0.49–0.88***	Not selected in the multivariate model		
Types of job										
Teaching	384	85.91	63	14.1		Ref			Ref	
Research	358	80.45	87	19.55	***0.030***	***1.48***	***1.03–2.11***	0.025	1.37	1.04–1.81
Admin	836	85.39	143	14.61	0.798	1.04	0.76–1.44	Not selected in the multivariate model		
Leadership	426	88.36	56	11.62	0.260	0.8	0.54–1.18			
Types of appointment										
Professional/Admin	778	85.68	130	14.32		Ref			Ref	
Academic (Teaching and/or Research)	1116	84.29	208	15.71	0.367	1.12	0.88–1.41	** < *0.001***	***2.45***	***1.78–3.37***
Leadership	106	92.17	9	7.83	0.06	0.51	0.26–1.02	Not selected in the multivariate model		
Levels of appointment										
Lecturer	293	84.2	55	15.8		Ref			Ref	
Senior Lecturer/Assistant Professor	394	87.36	57	12.64	0.202	0.77	0.52–1.15	Not selected in the multivariate model		
Associate Professor	188	93.07	14	6.93	***0.003***	***0.39***	***0.21–0.73***	0.023	0.51	0.29–0.91
Professor	215	89.58	25	10.42	0.063	0.61	0.37–1.02	Not selected in the multivariate model		
other	850	82.52	180	17.48	0.474	1.12	0.81–1.57	** < *0.001***	***2.66***	***1.93–3.68***
Current employment condition										
Full time	1665	86.49	260	13.51		Ref			Ref	
Part time	339	79.21	89	20.79	** < *0.001***	***1.68***	***1.29–2.19***	***0.003***	***1.53***	***1.15–2.02***
Self-reported co-morbidities										
No	1097	87.48	157	12.52		Ref			Ref	
Single co-morbidity	504	86.9	76	13.1	0.727	1.05	0.79–1.41	Not selected in the multivariate model		
Multiple co-morbidities	403	77.65	116	22.35	** < *0.001***	***2.01***	***1.54–2.62***	** < *0.001***	***1.86***	***1.41–2.48***
Self-reported co-morbidities										
No	1097	87.48	157	12.52		Ref			Ref	
Mental health issue	221	75.43	72	24.57	** < *0.001***	***2.28***	***1.66–3.12***	0.046	1.41	1.01–1.98
Other co-morbidity	686	85.11	120	14.89	0.125	1.22	0.95–1.58	Not selected in the multivariate model		
Current smoking										
Never smoker	1538	85.78	255	14.22		Ref		Not selected in the multivariate model		
Ever smoker (Daily, Non-daily, Ex)	466	83.21	94	16.79	0.137	1.22	0.94–1.58			
Increased smoking over the last 6 months										
No	75	85.23	13	14.77		Ref		Not selected in the multivariate model		
Yes	137	83.03	28	16.97	0.652	1.18	0.58–2.41			
Current alcohol drinking										
No	794	84.74	143	15.26		Ref		Not selected in the multivariate model		
Yes	1210	85.51	205	16.49	0.610	0.94	0.75–1.19			
Increased alcohol drinking over the last 6 months										
No	684	85.82	113	14.18		Ref		Not selected in the multivariate model		
Yes	109	78.99	29	21.01	***0.040***	***1.61***	***1.02–2.54***			
Physical activity for at least 30-min in the past week (days)										
None	409	83.64	80	16.36		Ref		Not selected in the multivariate model		
1–3 days	964	85.77	160	14.23	0.271	0.85	0.63–1.14			
4–7 days	629	85.46	107	14.54	0.385	0.87	0.63–1.19			
Experience related to COVID-19 pandemic										
No known exposure to COVID-19	1339	85.89	220	14.11		Ref		Not selected in the multivariate model		
Tested positive for COVID-19	663	83.82	128	16.18	0.182	1.18	0.93–1.49			
Symptoms of long COVID										
No	1379	86.79	229	14.24		Ref		Not selected in the multivariate model		
Yes	555	83.84	107	16.16	0.242	1.16	0.9–1.49			
Doses of COVID vaccine taken										
None	47	83.93	9	16.1		Ref		Not selected in the multivariate model		
Double	555	87.4	80	12.6	0.458	0.75	0.36–1.59			
Triple or more	1399	84.48	257	15.52	0.911	0.96	0.46–1.98			
Last checked/measured blood pressure										
Never checked blood pressure	141	81.03	33	18.97		Ref		Not selected in the multivariate model		
Checked within last 6 months	1227	85.8	203	14.2	0.095	0.71	0.47–1.06			
Checked > 6 months ago	559	85.34	96	14.66	0.165	0.73	0.47–1.13			
Last checked/measured blood lipid profile										
Never checked blood lipid profile	544	82.55	115	17.45		Ref			Ref	
Checked within last 6 months	697	86.8	106	13.2	***0.024***	***0.72***	***0.54–0.96***	***0.003***	***0.59***	***0.42–0.84***
Checked > 6 months ago	683	86.24	109	13.76	0.053	0.75	0.57–1.00	Not selected in the multivariate model		
Last checked/measured blood sugar										
Never checked blood sugar	397	82.88	82	17.12		Ref		Not selected in the multivariate model		
Checked within last 6 months	841	86.7	129	13.3	0.053	0.74	0.55–1.00			
Checked > 6 months ago	687	84.92	122	15.08	0.333	0.86	0.63–1.17			
Last visited a healthcare provider for general health assessment										
Never visited for general health assessment	327	85.6	55	14.4		Ref			Ref	
Visited within last 6 months	850	84.75	153	15.25	0.690	1.07	0.77–1.49	0.039	1.36	1.02–1.82
Visited > 6 months ago	753	85.76	125	14.24	0.940	0.99	0.70–1.39	Not selected in the multivariate model		
Perceived burnout (Burnout scale categories)										
No (score 1–4)	1485	89.24	179	10.76		Ref			Ref	
Yes (score 5–7)	515	75.29	169	24.71	** < *0.001***	***2.72***	***2.16–3.44***	** < *0.001***	***1.99***	***1.54–2.56***
Perceived status of own mental health										
Poor to Fair	385	73.06	142	26.94		Ref			Ref	
Good to Excellent	1576	88.89	197	11.11	** < *0.001***	***0.34***	***0.26–0.43***	** < *0.001***	***0.47***	***0.36–0.61***
Levels of psychological distress (K10 categories)										
Low (total score 10–15)	573	92.57	46	7.43		Ref			Ref	
Moderate to Very high (total score 16–50)	1392	82.61	293	17.39	** < *0.001***	***2.62***	***1.89–3.63***	***0.004***	***1.68***	***1.18–2.39***
Levels of coping (BRCS categories)										
Low resilient copers (score 4–13)	783	81.39	179	18.61		Ref		Not selected in the multivariate model		
Medium to High resilient copers (score 14–20)	1162	88.23	155	11.77	** < *0.001***	***0.58***	***0.46–0.74***			

^#^stepwise logistic regression method was applied, and variables were selected as significant level *p* < 0.05 in the univariate analyses and *p* < 0.01 in the multivariate analyses

### Burnout

Table 3 shows the associations between perceived burnout and other variables. After adjusting for potential confounders, burnout was associated with being female, having multiple co-morbidities, perceived job insecurity, and moderate to very high psychological distress. Conversely, staff with positive COVID-19 tests, recent blood sugar checks, and perceived mental health as good to excellent were less likely to report burnout.

**Table 3:** Predictors for perceived burnout among the study participants (based on the burnout scale)

Characteristics	No perceived burnout (score 1–4)		Perceived burnout (score 5–7)		Unadjusted analyses			Adjusted analyses#		
	n	%	n	%	*p*	ORs	95% CIs	*p*	AORs	95% CIs
Total study participants	1664		684							
Age groups										
18–29 years	164	72.25	63	27.75		Ref		Not selected in the multivariate model		
30–59 years	1351	70.55	564	29.45	0.595	1.09	0.80–1.48			
≥ 60 years	123	75	41	25	0.543	0.86	0.55–1.37			
Gender										
Male	654	74.91	219	25.1		Ref			Ref	
Female	988	68.8	448	31.2	***0.002***	***1.35***	***1.12–1.64***	***0.002***	***1.35***	***1.12–1.63***
Highest educational/vocational qualification										
Certificate/Diploma/Trade qualifications	140	66.99	69	33.01		Ref		Not selected in the multivariate model		
Bachelors level	385	72.37	147	27.63	0.147	0.77	0.55–1.09			
Masters level	534	70.63	222	29.37	0.309	0.84	0.61–1.17			
Doctoral level	592	70.81	244	29.19	0.280	0.84	0.60–1.16			
Duration of work										
< 5 years	378	73.54	136	26.46		Ref		Not selected in the multivariate model		
6–10 years	352	71.11	143	28.89	0.388	1.13	0.86–1.48			
> 10 years	897	69.59	392	30.41	0.096	1.22	0.97–1.53			
Current living status										
Live without family members (on your own/shared house)	259	68.16	121	31.84		Ref		Not selected in the multivariate model		
Live with family members (spouse/partners/siblings/children)	1383	71.25	558	28.75	0.226	0.86	0.68–1.10			
Types of job										
Teaching	326	73.59	117	26.41		Ref			Ref	
Research	322	72.52	122	27.48	0.720	1.06	0.78–1.42	Not selected in the multivariate model		
Admin	695	70.99	284	29.01	0.313	1.14	0.88–1.47			
Leadership	321	66.6	161	33.4	***0.021***	***1.40***	***1.05–1.86***	0.017	1.30	1.05–1.61
Types of appointment										
Professional/Admin	647	71.26	261	28.74		Ref			Ref	
Academic (Teaching and/or Research)	944	71.41	378	28.59	0.938	0.99	0.08–1.19	Not selected in the multivariate model		
Leadership	70	60.87	45	39.13	***0.023***	***1.59***	***1.07–2.38***	***0.021***	***1.59***	***1.07–2.36***
Levels of appointment										
Lecturer	251	72.33	96	27.67		Ref		Not selected in the multivariate model		
Senior Lecturer/Assistant Professor	308	68.44	142	31.56	0.234	1.21	0.88–1.64			
Associate Professor	150	74.26	52	25.74	0.624	0.91	0.61–1.34			
Professor	176	73.33	64	26.67	0.789	0.95	0.66–1.38			
Other	723	70.19	307	29.81	0.449	1.11	0.85–1.46			
Prefer not to say	45	71.43	18	28.57	0.883	1.05	0.58–1.90			
Current employment condition										
Full time	1363	70.99	557	29.01		Ref		Not selected in the multivariate model		
Part time	301	70.33	127	29.67	0.785	1.03	0.82–1.29			
Self-reported co-morbidities										
No	948	75.9	301	24.1		Ref			Ref	
Single co-morbidity	397	68.45	183	31.55	***0.001***	***1.45***	***1.17–1.81***	0.022	1.30	1.04–1.64
Multiple co-morbidities	319	61.46	200	38.45	** < *0.001***	***1.97***	***1.59–2.46***	***0.001***	***1.53***	***1.20–1.97***
Self-reported co-morbidities										
No	948	75.9	301	24.1		Ref			Ref	
Mental health issue	157	53.58	136	46.42	** < *0.001***	***2.73***	***2.10–3.55***	Not selected in the multivariate model		
Other co-morbidity	559	69.35	247	30.65	***0.001***	***1.39***	***1.14–1.69***	** < *0.001***	***1.87***	***1.41–2.48***
Current smoking										
Never smoker	1270	71.03	518	28.97		Ref		Not selected in the multivariate model		
Ever smoker (Daily, Non-daily, Ex)	394	70.36	166	29.64	0.760	1.03	0.84–1.27			
Increased smoking over the last 6 months										
No	48	54.55	40	45.45		Ref		Not selected in the multivariate model		
Yes	137	83,003	28	16.97	** < *0.001***	***0.25***	***0.14–0.44***			
Current alcohol drinking										
No	637	68.06	299	31.94		Ref		Not selected in the multivariate model		
Yes	1027	72.73	385	27.27	***0.015***	***0.79***	***0.67–0.96***			
Increased alcohol drinking over the last 6 months										
No	556	69.76	241	30.24		Ref		Not selected in the multivariate model		
Yes	81	58.7	57	41.3	***0.010***	***1.62***	***1.12–2.35***			
Physical activity for at least 30-min in the past week (days)										
None	313	64.14	175	35.86		Ref		Not selected in the multivariate model		
1–3 days	817	72.69	307	27.31	***0.001***	***0.67***	***0.54–0.84***			
4–7 days	533	72.62	201	27.38	***0.002***	***0.67***	***0.53–0.86***			
Experience related to COVID-19 pandemic										
No known exposure to COVID-19	1061	68.14	496	31.86		Ref			Ref	
Tested positive for COVID-19	602	76.3	187	23.7	** < *0.001***	***0.66***	***0.55–0.81***	** < *0.001***	***0.65***	***0.54–0.80***
Symptoms of long COVID										
No	1201	74.74	406	25.26		Ref			Ref	
Yes	407	61.48	255	38.52	** < *0.001***	***1.85***	***1.53–2.25***	Not selected in the multivariate model		
Doses of COVID vaccine taken										
None	42	75	14	25		Ref		Not selected in the multivariate model		
Double	463	73.03	171	26.97	0.750	1.11	0.59–2.07			
Triple or more	1156	69.89	498	30.11	0.413	1.29	0.69–2.38			
Last checked/measured blood pressure										
Never checked blood pressure	119	69.39	55	31.61		Ref		Not selected in the multivariate model		
Checked within last 6 months	1035	72.43	394	27.57	0.263	0.82	0.59–1.16			
Checked > 6 months ago	451	68.85	204	31.15	0.910	0.98	0.68–1.40			
Last checked/measured blood lipid profile										
Never checked blood lipid profile	455	69.04	204	30.96		Ref		Not selected in the multivariate model		
Checked within last 6 months	594	73.97	209	26.03	***0.037***	***0.78***	***0.62–0.99***			
Checked > 6 months ago	553	69.91	238	30.09	0.721	0.96	0.77–1.20			
Last checked/measured blood sugar										
Never checked blood sugar	333	69.52	146	30.48		Ref			Ref	
Checked within last 6 months	721	74.33	249	25.67	0.053	0.79	0.62–1.00	***0.004***	***0.76***	***0.63–0.92***
Checked > 6 months ago	550	68.07	258	31.93	0.588	1.07	0.84–1.37	Not selected in the multivariate model		
Last visited a healthcare provider for general health assessment										
Never visited for general health assessment	261	68.32	121	31.68		Ref		Not selected in the multivariate model		
Visited within last 6 months	723	72.16	279	27.84	0.160	0.83	0.64–1.08			
Visited > 6 months ago	624	71.15	253	28.85	0.313	0.87	0.67–1.13			
Job insecurity (JIS categories)										
No (total score 1–3)	1485	74.25	515	25.75		Ref			Ref	
Yes (total score 4–5)	179	51.44	169	48.56	** < *0.001***	***2.72***	***2.16–3.44***	** < *0.001***	***1.99***	***1.55–2.57***
Perceived status of own mental health										
Poor to Fair	246	46.86	279	53.14		Ref			Ref	
Good to Excellent	1385	78.12	388	21.88	** < *0.001***	***0.25***	***0.20–0.30***	** < *0.001***	***0.37***	***0.29–0.46***
Levels of psychological distress (K10 categories)										
Low (total score 10–15)	553	89.48	65	10.52		Ref			Ref	
Moderate to Very high (total score 16–50)	1080	64.17	603	35.83	** < *0.001***	***4.75***	***3.61–6.25***	** < *0.001***	***3.23***	***2.42–4.30***
Levels of coping (BRCS categories)										
Low resilient copers (score 4–13)	626	65.28	333	34.72		Ref		Not selected in the multivariate model		
Medium to High resilient copers (score 14–20)	991	75.25	326	24.75	** < *0.001***	***0.62***	***0.51–0.75***			

^*#*^stepwise logistic regression method was applied, and variables were selected as significant level *p* < 0.05 in the univariate analyses and *p* < *0.01* in the multivariate analyses

### Psychological distress

Univariate analyses showed that moderate to very high psychological distress was associated with having single or multiple co-morbidities, mental health issues, smoking, perceived job insecurity and burnout (Table 4). Conversely, lower distress levels were reported by staff aged ≥ 60 years, holding Senior Lecturer/Assistant Professor or Professor appointments, perceiving good to excellent mental health, and reporting medium to high resilient coping.

**Table 4:** Predictors for higher psychological distress among the study participants (based on the K-10 scale)

Characteristics	Low psychological distress (total score 10–15)		Moderate to Very high psychological distress (total score 16–50)		Unadjusted analyses			Adjusted analyses#		
	n	%	n	%	*p*	ORs	95% CIs	*p*	AORs	95% CIs
Total study participants	619		1685							
Age groups										
18–29 years	43	19.37	179	80.63		Ref			Ref	
30–59 years	489	26.1	1386	73.92	***0.031***	***0.69***	***0.48–0.97***	0.034	0.69	0.48–0.97
≥ 60 years	71	43.29	93	56.71	** < *0.001***	***0.31***	***0.20–0.50***	** < *0.001***	***0.31***	***0.20–0.50***
Gender										
Male	260	30.13	603	69.87		Ref			Ref	
Female	353	25.12	1052	74.88	***0.009***	***1.28***	***1.06–1.55***	Not selected in the multivariate model		
Highest educational/vocational qualification										
Certificate/Diploma/Trade qualifications	54	26.1	153	73.91		Ref		Not selected in the multivariate model		
Bachelors level	129	24.76	392	75.24	0.710	1.07	0.74–1.55			
Masters level	189	25.27	559	74.73	0.811	1.04	0.73–1.48			
Doctoral level	245	30.14	568	69.86	0.254	0.82	0.58–1.15			
Duration of work										
< 5 years	126	25.05	377	74.95		Ref		Not selected in the multivariate model		
6–10 years	123	25.52	359	74.48	0.866	0.98	0.73–1.30			
> 10 years	358	28.21	911	71.79	0.178	0.85	0.67–1.08			
Current living status										
Live without family members (on your own/shared house)	85	22.73	289	77.27		Ref		Not selected in the multivariate model		
Live with family members (spouse/partners/siblings/children)	527	27.71	1375	72.29	***0.048***	***0.77***	***0.59–0.99***			
Types of job										
Teaching	132	30.28	304	69.72		Ref			Ref	
Research	116	26.61	320	73.39	0.230	1.19	0.99–1.61	Not selected in the multivariate model		
Admin	234	24.38	726	75.62	***0.020***	***1.35***	***1.05–1.73***	0.019	1.26	1.04–1.52
Leadership	137	29.03	335	70.97	0.680	1.06	0.79–1.41	Not selected in the multivariate model		
Types of appointment										
Professional/Admin	217	24.3	676	75.7		Ref		Not selected in the multivariate model		
Academic (Teaching and/or Research)	376	29.1	917	70.92	***0.014***	***0.78***	***0.64–0.95***			
Leadership	26	23.01	87	76.99	0.763	1.07	0.68–1.71			
Levels of appointment										
Lecturer	82	24.26	256	75.74		Ref			Ref	
Senior Lecturer/Assistant Professor	149	33.79	292	66.21	***0.004***	***0.63***	***0.45–0.86***	** < *0.001***	***0.58***	***0.46–0.74***
Associate Professor	60	30.61	136	69.39	0.110	0.73	0.49–1.07	0.015	0.66	0.47–0.92
Professor	83	34.73	156	65.27	***0.006***	***0.6***	***0.41–0.87***	** < *0.001***	***0.55***	***0.41–0.74***
Other	233	22.98	781	77.02	0.629	1.07	0.80–1.43			
Prefer not to say	9	15.25	50	84.75	0.133	1.77	0.84–3.77			
Current employment condition										
Full time	504	26.72	1382	73.28		Ref		Not selected in the multivariate model		
Part time	115	27.51	303	72.49	0.742	0.96	0.76–1.22			
Self-reported co-morbidities										
No	382	31.13	845	68.87		Ref			Ref	
Single co-morbidity	137	24.25	428	75.75	***0.003***	***1.41***	***1.13–1.77***	0.037	1.29	1.01–1.63
Multiple co-morbidities	100	19.53	412	80.47	** < *0.001***	***1.87***	***1.45–2.39***	***0.006***	***1.46***	***1.11–1.92***
Self-reported co-morbidities										
No	382	31.13	845	68.87		Ref			Ref	
Mental health issue	31	10.76	257	89.24	** < *0.001***	***3.75***	***2.53–5.54***	** < *0.001***	***2.73***	***1.79–4.15***
Other co-morbidity	206	26.11	853	73.89	***0.016***	***1.28***	***1.05–1.56***			
Current smoking										
Never smoker	491	27.96	1265	72.04		Ref			Ref	
Ever smoker (Daily, Non-daily, Ex)	128	23.36	420	76.64	***0.034***	***1.27***	***1.02–1.59***	0.029	1.29	1.03–1.61
Increased smoking over the last 6 months										
No	11	12.79	75	87.21		Ref		Not selected in the multivariate model		
Yes	33	20.62	127	79.38	0.130	0.56	0.26–1.18			
Current alcohol drinking										
No	250	27.32	665	72.68		Ref		Not selected in the multivariate model		
Yes	369	26.57	1020	73.43	0.689	1.04	0.86–1.25			
Increased alcohol drinking over the last 6 months										
No	240	30.77	540	69.23		Ref		Not selected in the multivariate model		
Yes	10	7.52	123	72.48	** < *0.001***	***5.47***	***2.82–10.6***			
Physical activity for at least 30-min in the past week (days)										
None	101	21.13	377	78.87		Ref		Not selected in the multivariate model		
1–3 days	281	25.41	825	74.59	0.068	0.79	0.60–1.01			
4–7 days	236	32.87	482	67.13	** < *0.001***	***0.55***	***0.42–0.72***			
Experience related to COVID-19 pandemic										
No known exposure to COVID-19	387	25.34	1140	74.66		Ref			Ref	
Tested positive for COVID-19	232	29.94	543	70.06	***0.019***	***0.79***	***0.66–0.96***	***0.012***	***0.78***	***0.64–0.95***
Symptoms of long COVID										
No	487	30.94	1087	69.06		Ref		Not selected in the multivariate model		
Yes	117	18	533	82	** < *0.001***	***2.04***	***1.62–2.56***			
Doses of COVID vaccine taken										
None	14	26.92	38	73.08		Ref		Not selected in the multivariate model		
Double	159	25.6	462	74.4	0.834	1.07	0.57–2.03			
Triple or more	445	27.37	1181	72.63	0.944	0.98	0.52–1.82			
Last checked/measured blood pressure										
Never checked blood pressure	43	24.71	131	75.29		Ref		Not selected in the multivariate model		
Checked within last 6 months	404	28.25	1026	71.75	0.326	0.83	0.58–1.19			
Checked > 6 months ago	164	25.04	491	74.96	0.930	0.98	0.67–1.45			
Last checked/measured blood lipid profile										
Never checked blood lipid profile	162	24.58	497	75.42		Ref		Not selected in the multivariate model		
Checked within last 6 months	232	28.89	571	71.11	0.065	0.8	0.63–1.01			
Checked > 6 months ago	217	27.4	575	72.6	0.224	0.86	0.68–1.10			
Last checked/measured blood sugar										
Never checked blood sugar	124	25.89	355	74.11		Ref			Ref	
Checked within last 6 months	288	29.69	682	70.31	0.131	0.82	0.65–1.06	0.038	1.23	1.01–1.50
Checked > 6 months ago	198	24.47	611	75.53	0.572	1.08	0.83–1.40	Not selected in the multivariate model		
Last visited a healthcare provider for general health assessment										
Never visited for general health assessment	99	25.92	283	74.08		Ref		Not selected in the multivariate model		
Visited within last 6 months	275	27.42	728	72.58	0.574	0.93	0.71–1.21			
Visited > 6 months ago	239	27.22	639	72.78	0.631	0.93	0.71–1.23			
Job insecurity (JIS categories)										
No (total score 1–3)	573	29.16	1392	70.8		Ref			Ref	
Yes (total score 4–5)	46	13.57	293	86.43	** < *0.001***	***2.62***	***1.89–3.63***	***0.009***	***1.61***	***1.13–2.30***
Perceived burnout (Burnout scale categories)										
No (score 1–4)	553	33.86	1080	66.14		Ref			Ref	
Yes (score 5–7)	65	9.73	603	90.27	** < *0.001***	***4.75***	***3.61–6.25***	** < *0.001***	***3.22***	***2.41–4.31***
Perceived status of own mental health										
Poor to Fair	8	1.52	519	98.48		Ref			Ref	
Good to Excellent	609	34.35	1164	65.65	** < *0.001***	***0.03***	***0.01–0.05***	** < *0.001***	***0.05***	***0.02–0.10***
Levels of coping (BRCS categories)										
Low resilient copers (score 4–13)	149	15.49	813	84.51		Ref			Ref	
Medium to High resilient copers (score 14–20)	467	35.46	850	64.54	** < *0.001***	***0.33***	***0.27–0.41***	** < *0.001***	***0.48***	***0.39–0.60***

^#^stepwise logistic regression method was applied and variables were selected as significant level* p* < 0.05 in the univariate analyses and *p* < 0.01 in the multivariate analyses

### Coping

Table 5 shows the factors associated with medium to high resilient coping. Staff with Bachelor and higher qualifications, Professor appointments, smoking, current alcohol use, recent blood lipid profile checks, and perceived good to excellent mental health were more likely to exhibit medium to high resilient coping. Conversely, staff in administration or leadership roles, with single co-morbidity, mental health issues, and moderate to very high psychological distress showed low resilient coping levels.

**Table 5:** Predictors for high resilient coping among the study participants (based on the BRCS scale)

Characteristics	Low resilient copers (score 4–13)		Medium to High resilient copers (score 14–20)		Unadjusted analyses			Adjusted analyses#		
	n	%	n	%	*p*	ORs	95% CIs	*p*	AORs	95% CIs
Total study participants	962		1317							
Age groups										
18–29 years	89	40.8	129	59.17		Ref		Not selected in the multivariate model		
30–59 years	791	42.66	1063	57.34	0.603	0.92	0.69–1.23			
≥ 60 years	58	35.37	106	64.63	0.278	1.26	0.83–1.92			
Gender										
Male	331	38.71	524	61.29		Ref		Not selected in the multivariate model		
Female	611	43.99	778	56.01	***0.014***	***0.80***	***0.68–0.96***			
Highest educational/vocational qualification										
Certificate/Diploma/Trade qualifications	112	54.63	93	45.37		Ref			Ref	
Bachelors level	216	41.94	299	58.06	***0.002***	***1.67***	***1.20–2.30***	***0.001***	***1.71***	***1.23–2.38***
Masters level	291	39.27	450	60.73	** < *0.001***	***1.86***	***1.36–2.54***	** < *0.001***	***1.89***	***1.38–2.59***
Doctoral level	334	41.59	469	58.41	***0.001***	***1.69***	***1.24–2.30***	***0.001***	***1.69***	***1.24–2.31***
Duration of work										
< 5 years	192	38.71	304	61.29		Ref		Not selected in the multivariate model		
6–10 years	198	41.42	280	58.58	0.388	0.89	0.69–1.15			
> 10 years	546	43.51	709	56.49	0.067	0.82	0.66–1.01			
Current living status										
Live without family members (on your own/shared house)	155	41.67	217	58.33		Ref		Not selected in the multivariate model		
Live with family members (spouse/partners/siblings/children)	797	42.42	1082	57.58	0.789	0.97	0.77–1.22			
Types of job										
Teaching	157	36.43	274	63.57		Ref			Ref	
Research	171	39.49	262	60.51	0.353	0.88	0.67–1.16	Not selected in the multivariate model		
Admin	427	44.85	525	55.15	***0.003***	***0.70***	***0.56–0.89***	***0.002***	***0.75***	***0.62–0.92***
Leadership	207	44.71	256	55.29	***0.012***	***0.71***	***0.54–0.93***	***0.003***	***0.73***	***0.58–0.92***
Types of appointment										
Professional/Admin	390	44.17	493	55.83		Ref		Not selected in the multivariate model		
Academic (Teaching and/or Research)	532	41.5	750	58.5	0.217	1.12	0.94–1.33			
Leadership	37	33.94	72	66.06	***0.043***	***1.54***	***1.01–2.34***			
Levels of appointment										
Lecturer	154	46.39	178	53.61		Ref			Ref	
Senior Lecturer/Assistant Professor	173	39.41	266	60.59	0.052	1.33	0.99–1.77	***0.043***	***1.26***	***1.01–1.59***
Associate Professor	78	40	117	60	0.154	1.29	0.91–1.86	Not selected in the multivariate model		
Professor	64	27.12	172	72.88	** < *0.001***	***2.33***	***1.62–3.33***	***0.001***	***2.24***	***1.64–3.05***
Other	452	45	551	54.94	0.675	1.05	0.82–1.35	Not selected in the multivariate model		
Not to say	30	52.63	27	47.37	0.384	0.78	0.44–1.37			
Current employment condition										
Full time	791	42.41	1074	57.59		Ref		Not selected in the multivariate model		
Part time	171	41.3	243	58.7	0.68	1.04	0.84–1.29			
Self-reported co-morbidities										
No	446	36.77	767	63.23		Ref			Ref	
Single co-morbidity	263	46.88	298	53.12	** < *0.001***	***0.66***	***0.54–0.81***	***0.004***	***0.73***	***0.59–0.91***
Multiple co-morbidities	253	50.1	252	49.39	** < *0.001***	***0.58***	***0.47–0.71***	***0.016***	***0.74***	***0.59–0.95***
Self-reported co-morbidities										
No	446	36.77	767	63.23		Ref			Ref	
Mental health issue	180	63.16	105	36.84	** < *0.001***	***0.34***	***0.26–0.44***	** < *0.001***	***0.46***	***0.34–0.61***
Other co-morbidity	336	43.02	445	56.98	***0.005***	***0.77***	***0.64–0.93***	Not selected in the multivariate model		
Current smoking										
Never smoker	754	43.43	982	56.57		Ref			Ref	
Ever smoker (Daily, Non-daily, Ex)	208	38.31	335	61.69	***0.035***	***1.24***	***1.02–1.51***	***0.007***	***1.33***	***1.09–1.63***
Increased smoking over the last 6 months										
No	27	31.4	59	68.6		Ref		Not selected in the multivariate model		
Yes	58	36.25	102	63.75	0.446	0.81	0.46–1.41			
Current alcohol drinking										
No	450	49.61	457	50.39		Ref			Ref	
Yes	512	37.32	860	62.68	** < *0.001***	***1.65***	***1.39–1.96***	** < *0.001***	***1.61***	***1.35–1.92***
Increased alcohol drinking over the last 6 months										
No	370	47.93	402	52.07		Ref		Not selected in the multivariate model		
Yes	78	58.65	55	41.32	***0.023***	***0.65***	***0.45–0.94***			
Physical activity for at least 30-min in the past week (days)										
None	190	40.34	281	59.66		Ref		Not selected in the multivariate model		
1–3 days	464	42.34	632	57.66	0.463	0.92	074–1.14			
4–7 days	306	43.1	404	56.9	0.347	0.89	0.70–1.13			
Experience related to COVID-19 pandemic										
No known exposure to COVID-19	668	44.24	842	55.76		Ref			Ref	
Tested positive for COVID-19	293	38.2	474	61.8	***0.006***	***1.28***	***1.07–1.53***	0.040	1.21	1.01–1.46
Symptoms of long COVID										
No	645	41.43	912	58.57		Ref		Not selected in the multivariate model		
Yes	281	43.7	362	56.3	0.326	0.91	0.76–1.10			
Doses of COVID vaccine taken										
None	14	26.92	38	73.08		Ref		Not selected in the multivariate model		
Double	258	41.75	360	58.25	***0.039***	***0.51***	***0.27–0.97***			
Triple or more	687	42.83	917	57.17	***0.025***	***0.49***	***0.26–0.91***			
Last checked/measured blood pressure										
Never checked blood pressure	88	50.57	86	49.43		Ref		Not selected in the multivariate model		
Checked within last 6 months	570	39.86	860	60.14	***0.007***	***1.54***	***1.13–2.12***			
Checked > 6 months ago	293	44.73	362	55.27	0.170	1.26	0.90–1.77			
Last checked/measured blood lipid profile										
Never checked blood lipid profile	338	51.29	321	48.71		Ref			Ref	
Checked within last 6 months	280	340.87	523	65.13	** < *0.001***	***1.97***	***1.59–2.43***	** < *0.001***	***1.86***	***1.51–2.30***
Checked > 6 months ago	330	41.67	462	58.33	** < *0.001***	***1.47***	***1.19–1.82***	***0.001***	***1.42***	***1.15–1.76***
Last checked/measured blood sugar										
Never checked blood sugar	245	51.15	234	48.385		Ref		Not selected in the multivariate model		
Checked within last 6 months	360	37.11	610	62.89	** < *0.001***	***1.77***	***1.42–2.21***			
Checked > 6 months ago	345	42.65	464	57.35	***0.003***	***1.41***	***1.12–1.77***			
Last visited a healthcare provider for general health assessment										
Never visited for general health assessment	196	51.31	186	48.69		Ref		Not selected in the multivariate model		
Visited within last 6 months	382	38.09	621	61.91	** < *0.001***	***1.71***	***1.35–2.17***			
Visited > 6 months ago	376	42.82	502	57.18	***0.006***	***1.41***	***1.11–10.8***			
Job insecurity (JIS categories)										
No (total score 1–3)	783	40.26	1162	59.74		Ref		Not selected in the multivariate model		
Yes (total score 4–5)	179	53.59	155	46.41	** < *0.001***	***0.58***	***0.46–0.74***			
Perceived burnout (Burnout scale categories)										
No (score 1–4)	626	38.71	991	61.29		Ref		Not selected in the multivariate model		
Yes (score 5–7)	333	50.53	326	49.47	** < *0.001***	***0.62***	***0.52–0.74***			
Perceived status of own mental health										
Poor to Fair	356	68.86	161	31.14		Ref			Ref	
Good to Excellent	603	34.3	1155	65.7	** < *0.001***	***4.23***	***3.43–5.22***	** < *0.001***	***3.36***	***2.69–4.19***
Levels of psychological distress (K10 categories)										
Low (total score 10–15)	149	24.19	467	75.81		Ref			Ref	
Moderate to Very high (total score 16–50)	813	48.89	850	51.11	** < *0.001***	***0.344***	***0.27–0.41***	** < *0.001***	***0.47***	***0.38–0.58***

^#^stepwise logistic regression method was applied and variables were selected as significant level *p* < 0.05 in the univariate analyses and *p* < 0.01 in the multivariate analyses

### Country-wise findings

Country-wise analyses showed varied proportions of job insecurity, burnout, psychological distress, and coping across all 16 countries. Details are included in Table 6.

**Table 6:** Country-wise analyses for job insecurity, perceived burnout, higher psychological distress and high resilient coping among the study participants

Characteristics	JIS scale				Unadjusted analyses			Adjusted analyses#		
	No job insecurity (total score 1–3)		Job insecurity (total score 4–5)							
	n	%	n	%	*p*	ORs	95% CIs	*p*	AORs	95% CIs
Country/area of residence										
Turkey (Lowest prevalence category)	138	93.88	9	6.12		Ref			Ref	
Indonesia	57	93.44	4	6.56	0.906	1.08	0.32–3.63	Not selected in the multivariate model		
Kuwait	87	90.62	9	6.56	0.347	1.59	0.61–4.15			
Oman	79	91.86	7	8.14	0.558	1.36	0.49–3.79			
Malaysia	142	91.61	13	8.39	0.451	1.40	0.58–3.39			
Taiwan, China	42	91.30	4	8.70	0.545	1.46	0.43–4.98			
Palestine	83	91.21	8	8.79	0.44	1.48	0.55–3.97			
United Kingdom	308	89.53	36	10.47	0.131	1.79	0.84–3.82			
Mexico	282	89.24	34	10.76	0.114	1.84	0.86–3.96			
South Africa	55	84.62	10	15.38	***0.035***	***2.78***	***1.07–7.23***			
Mainland China	115	83.94	22	16.06	***0.010***	***2.93***	***1.29–6.62***			
Hong Kong, China	153	82.26	33	17.74	***0.002***	***3.31***	***1.52–7.16***			
Pakistan	30	81.08	7	18.92	***0.019***	***3.58***	***1.24–10.4***	0.011	3.08	1.29–7.38
Saudi Arabia	36	76.60	11	23.40	***0.002***	***4.69***	***1.80–12.2***	***0.003***	***3.23***	***1.48–7.03***
United Arab Emirates	86	74.78	29	25.22	** < *0.001***	***5.17***	***2.34–11.5***	** < *0.001***	***3.11***	***1.91–5.08***
Australia	311	73.35	113	26.65	** < *0.001***	***5.57***	***2.75–11.3***	** < *0.001***	***2.38***	***1.76–3.21***
Characteristics	Burnout scale				Unadjusted analyses			Adjusted analyses#		
	No perceived burnout (score 1–4)		Perceived burnout (score 5–7)							
	n	%	n	%	*p*	ORs	95% CIs	*p*	AORs	95% CIs
Country/area of residence										
] Pakistan (Lowest prevalence category)	36	97.30	1	2.70		Ref			Ref	
Indonesia	53	86.89	8	13.11	0.118	5.43	0.66–45.3	Not selected in the multivariate model		
Turkey	121	82.31	26	17.69	***0.048***	***7.74***	***1.01–59.0***			
Oman	68	79.07	18	20.93	***0.031***	***9.52***	***1.22–74.3***	***0.009***	***2.51***	***1.25–5.04***
Malaysia	121	78.06	34	21.94	***0.025***	***10.1***	***1.34–76.5***	** < *0.001***	***2.77***	***1.58–4.82***
United Kingdom	261	76.32	81	23.68	***0.018***	***11.7***	***1.51–82.8***	***0.002***	***2.10***	***1.21–3.37***
Hong Kong, China	135	72.97	50	27.03	***0.012***	***13.3***	***1.78–99.8***	***0.010***	***2.01***	***1.18–3.41***
Kuwait	69	71.88	27	28.12	***0.011***	***14.1***	***10.8–108***	** < *0.001***	***3.81***	***2.05–7.07***
Taiwan, China	33	71.74	13	28.26	***0.013***	***14.2***	***1.76–114***	0.014	2.77	1.23–6.23
Saudi Arabia	33	70.21	14	29.79	***0.010***	***15.3***	***1.90–123***	***0.001***	***3.99***	***1.83–8.72***
Palestine	63	69.23	28	30.77	***0.008***	***16.0***	***2.09–123***	** < *0.001***	***3.96***	***2.14–7.35***
United Arab Emirates	79	68.70	36	31.30	***0.007***	***16.4***	***2.16–124***	** < *0.001***	***3.81***	***2.11–6.88***
Mexico	214	67.72	102	32.28	***0.005***	***17.2***	***2.32–127***	** < *0.001***	***5.31***	***3.27–8.62***
South Africa	42	64.62	23	35.38	***0.004***	***19.7***	***2.53–153***	***0.001***	***3.38***	***1.69–6.78***
Mainland China	86	62.77	51	37.23	***0.003***	***21.4***	***2.84–161***	** < *0.001***	***3.51***	***2.02–6.11***
Australia	250	59.24	172	40.76	***0.002***	***24.8***	***3.36–182***	** < *0.001***	***5.27***	***3.34–8.31***
Characteristics	K-10 scale				Unadjusted analyses			Adjusted analyses#		
	Low psychological distress (total score 10–15)		Moderate to Very high psychological distress (total score 16–50)							
	n	%	n	%	*p*	ORs	95% CIs	*p*	AORs	95% CIs
Country/area of residence										
Oman (Lowest prevalence category)	33	39.29	51	60.71		Ref			Ref	
Mexico	106	34.42	202	65.58	0.410	1.23	0.75–2.02	Not selected in the multivariate model		
Pakistan	12	33.33	24	66.67	0.538	1.29	0.57–2.93			
United Kingdom	113	33.24	227	66.76	0.297	1.29	0.79–2.12	** < *0.001***	***0.55***	***0.40–0.76***
Kuwait	29	30.53	66	69.47	0.220	1.47	0.79–2.73	Not selected in the multivariate model		
Saudi Arabia	14	30.43	32	69.57	0.316	1.47	0.68–3.18			
Australia	116	28.36	293	71.64	***0.048***	***1.63***	***1.00–2.66***	** < *0.001***	***0.46***	***0.33–0.62***
United Arab Emirates	32	28.07	82	71.93	0.098	1.66	0.91–3.01	Not selected in the multivariate model		
Taiwan, China	12	27.27	32	72.73	0.179	1.73	0.78–3.82			
Malaysia	35	22.73	119	77.27	***0.007***	***2.20***	***1.23–3.92***			
South Africa	14	22.22	49	77.78	***0.030***	***2.26***	***1.09–4.74***			
Indonesia	13	21.67	47	78.33	***0.027***	***2.33***	***1.10–4.97***	0.049	1.99	1.00–3.94
Palestine	18	20.00	72	80.00	***0.006***	***2.59***	***1.32–5.10***	0.011	2.12	1.18–3.79
Mainland China	25	18.52	110	81.48	***0.001***	***2.85***	***1.54–5.27***	Not selected in the multivariate model		
Hong Kong, China	30	16.76	149	83.24	** < *0.001***	***3.21***	***1.78–5.78***	0.045	1.62	1.01–2.60
Turkey	17	11.56	130	88.44	** < *0.001***	***4.94***	***2.54–9.66***	** < *0.001***	***3.96***	***20.3–6.95***
Characteristics	BRCS scale				Unadjusted analyses			Adjusted analyses#		
	Low resilient copers (score 4–13)		Medium to High resilient copers (score 14–20)							
	n	%	n	%	*p*	ORs	95% CIs	*p*	AORs	95% CIs
Country/area of residence										
Australia (Lowest prevalence category)	240	60	160	40.00		Ref			Ref	
United Kingdom	199	59.05	138	40.95	0.794	1.04	0.77–1.39	Not selected in the multivariate model		
South Africa	36	57.14	27	42.86	0.668	1.13	0.66–1.93			
Hong Kong, China	90	50.56	88	49.44	***0.035***	***1.47***	***1.03–2.10***			
Malaysia	76	49.67	77	50.33	***0.029***	***1.52***	***1.04–2.21***			
Kuwait	45	47.37	50	52.63	***0.026***	***1.67***	***1.06–2.61***			
United Arab Emirates	44	39.64	67	60.36	** < *0.001***	***2.28***	***1.49–3.51***			
Saudi Arabia	17	36.96	29	63.04	***0.004***	***2.55***	***1.36–4.81***			
Pakistan	12	34.29	23	65.71	***0.004***	***2.88***	***1.39–5.94***			
Mainland China	41	31.06	91	68.94	** < *0.001***	***3.33***	***2.18–5.06***	** < *0.001***	***3.15***	***2.04–4.83***
Oman	26	30.95	58	69.05	** < *0.001***	***3.35***	***2.02–5.53***	0.018	1.89	1.12–3.19
Palestine	326	28.89	64	71.11	** < *0.001***	***3.69***	***2.24–60.1***	***0.001***	***2.34***	***1.41–3.87***
Indonesia	17	28.81	42	71.19	** < *0.001***	***3.71***	***2.04–6.74***	***0.003***	***2.67***	***1.38–5.15***
Turkey	31	21.23	115	78.77	** < *0.001***	***5.57***	***3.57–8.68***	** < *0.001***	***6.59***	***2.43–10.3***
Taiwan, China	9	20.45	35	79.55	** < *0.001***	***5.83***	***2.73–12.5***	** < *0.001***	***5.85***	***2.63–13.0***
Mexico	53	17.32	253	82.68	** < *0.001***	***7.16***	***5.01–10.2***	** < *0.001***	***5.42***	***3.85–7.63***

^#^stepwise logistic regression method was applied and variables were selected as significant level *p* < 0.05 in the univariate analyses and *p* < 0.01 in the multivariate analyses

## Discussion

This is the first large-scale cross-sectional global study examining the health and wellbeing of staff working in HE institutions during the post-pandemic period. The study assessed job insecurity, burnout, psychological distress, and coping difficulties amongst HE staff in 16 countries, and identified the sub-groups at high-risk of experiencing difficulties. This study was conducted within the post-pandemic environment, when the environment at HE institutions was a bit unstable with financial challenges and restructuring of operations, therefore, challenges faced by the staff during that period were extraordinary. Compared to the pre-pandemic and pandemic contexts, the impact was heightened during the post-pandemic period. Therefore, findings from this study add critical insights for relevant support policies for wellbeing of staff working at HE institutions which should be adopted in the strategic directions, so that the environment can be more productive, resilient and sustainable to face any future challenges.

### Job insecurity

Job insecurity, a personal concern about future employment stability, is a growing concern in public and private universities [19, 20]. Financial pressures from pandemic-induced technological advancements, the complexities of teaching, research and societal contributions, underpin uncertainty about tenured employment [21, 22]. However, 85% of staff in this study did not perceive job insecurity, contrasting sharply with findings from a 2021 study of academics in Australian universities. In that study, 77% feared job loss, 50% were concerned about damaged career prospects, and 81% predicted increased casualisation [23]. Similar findings were reported in studies conducted elsewhere [24].

Our findings may be explained by the post-pandemic period in 2023, during which cost-cutting measures, including extensive job redundancies, were implemented. HE institutions navigated organizational changes and moved forward[25, 26], as evidenced by a study of the impact of restructuring during the COVID-19 pandemic. The impact of COVID-related job insecurity, and the global financial crisis may have varied across sectors, such as hospitality or industry, compared to higher education.

Job insecurity was significantly associated with staff in research roles or with academic appointments (teaching and/or research). This is consistent with research identifying job insecurity as a direct stressor, particularly in research work [27]; and studies showing that higher levels of education could contribute to less job insecurity [28]. The findings might also be attributed to increased workloads, work demands, and high expectations around performance in HE institutions [29]. Part-time employment but not the duration of work (working ≥ 10 years) significant predicted job insecurity, and this finding is consistent with studies showing that job tenure significantly predicts job insecurity; although increasingly, universities are transitioning away from tenured positions [22].

A review showed that teachers with temporary contracts of < 3 months had the highest levels of depression and anxiety and fear of job loss [29]. Our study also showed that staff with multiple co-morbidities or mental health issues or moderate to high levels of psychological distress had higher levels of job insecurity. The emergence of COVID-19 had a significant effect on the psychological wellbeing of HE employees during the pandemic [21]. Perceived job insecurity has been linked to deterioration of health and well-being as it increases anxiety, worry, and depression [19, 27, 30]. Furthermore, perceived burnout was also associated with higher job insecurity in this study, consistent with prior research identifying job insecurity as a chronic stress reaction stemming from an inability to cope adaptively in stressful situations [31].

### Burnout

Similar to previous research, one-third of staff reported perceived burnout, with women reporting higher levels than men [32, 33]. Explanations may include household responsibilities, parenting, unfulfilled motivations for research productivity, work-life balance challenges, and lack of support [33, 34].

Staff with leadership roles experienced higher levels of burnout. In a 2022 USA study of health sciences faculty, participants reported medium to high levels of burnout, and, like this study, most participants were women [34]. Contributing factors included campus closures, limited preparation for transitioning to new learning modalities, ongoing planning for reopening, government mandates, and additional workloads and responsibilities [33, 35, 36]. Further, a recent Australian university study (2023) of Nursing and Allied Health staff reported increased workload and burnout, and identified several contributing factors, including managing distressed students, online teaching, stepping into leadership roles, staff shortages, and sourcing placements [37].

Poor mental health among HE staff, especially among women has been highlighted previously with recommendations for realistic workload allocations, better performance indicators, long-term goal setting for academics and sustainable career pathways [32, 33].

### Psychological distress

The present study showed that more than two-thirds of participants experienced moderate to very high levels of psychological distress. Although numerous studies have investigated mental health issues among university students, the exploration of psychological distress in HE institutions involving university administrative staff, academics, and other support personnel in the post-pandemic period is limited.

The scarcity of similar university staff-focused research globally constraints meaningful comparisons with previous studies. However, our study showed comparatively higher rates of psychological distress (73%) compared to other studies, which varied between 9%-54% [38–40]. A longitudinal study focusing on teachers and staff affiliated with a Japanese university reported a significant increase in psychological distress in 2021 compared to 2019 [41]. A study from a South African university showed that 28% of staff experienced psychological distress, with administrative and service staff experiencing higher distress compared to academic staff [42]. In contrast, the current study included all university staff (academics, researchers, administrative and other support staff) across different HE institutions globally.

In this study, psychological distress varied according to age and gender [40–42]. Younger staff were at risk of experiencing higher levels of psychological distress; a finding also supported by previous research where younger age was identified as a risk factor for mental health issues [38, 41]. Younger individuals may be more exposed to social media, potentially increasing their exposure to pandemic-related negative and misleading news, thereby exacerbating stress [16, 43]. Young people may also experience distress due to their inherent responsibility for social productivity and family maintenance [44].

Higher levels of psychological distress were experienced by women compared to men; similar findings have been reported in Italy [40], Japan [41], and South Africa [42]. Academic women faced more challenges during the pandemic, primarily because of the greater load in terms of household chores, family care responsibilities, and providing emotional support [45]. Further, both young adults and women experience higher levels of anxiety due to frequent exposed to unemployment and economic risks, leading to increased psychological distress [46].

Staff reporting any co-morbidity and mental health issues also had higher levels of psychological distress, and this finding was supported by recent studies showing an association between perceived poor mental health and higher levels of psychological distress [38, 42]. Another study reported an increase in psychological distress among staff with a history of psychiatric treatment [47].

### Coping

Coping refers to cognitive and behavioural mechanisms that enhance resilience to stress. Strategies fall into higher order dimensions: adaptive/maladaptive, avoidant/approach, problem, emotional or avoidance-focused coping [48]. In our study over half the staff coped adaptively, with normal to high resilience scores on the BRCS. Similar results were reported in a large international sample during COVID-19 [9]. An Australian study also found that individuals with higher qualifications (i.e., bachelor’s degree) reported increased normal to high resilience scores [49]. This was attributed to enhanced critical thinking and problem-solving skills leading to the use of more adaptive coping strategies. Overall, cognitive reappraisal, social support and active coping are adaptive coping strategies used for managing stress and maintaining wellbeing. Emotion suppression has mixed effects [50]. Problem focused coping strategies such as seeking support, acceptance, exercise, and leisure activities are adaptive and link to increased wellbeing, while avoidance and emotion-focused coping are maladaptive and associated with lower levels of wellbeing [51–54].

Surprisingly, participants classified as normal/high resilient copers were more likely to smoke and consume alcohol, consistent with findings among academic staff in Malaysia and Saudi Arabi during the pandemic [51, 52]. Additionally, the resilient copers were more likely to report their mental health as excellent, consistent with previous findings among academics from the UAE [51] and Malaysia [52]. This supports the notion that adaptive problem focused coping strategies (normal/high resilience) are associated with enhanced mental health and wellbeing, while emotion focused coping strategies (low resilience) are more likely to be maladaptive and have an inverse relationship with mental health and wellbeing [55]. For example, an Australian study concluded that low resilient copers were associated with multiple comorbidities impairing their ability to adopt healthy behaviours over time [16].

### Country-wise analyses

The impact of COVID-19 and issues around professional workloads had varying impacts on staff health and wellbeing across the participating countries. Nevertheless, some trends were observed in some countries. For example, participants from the two Australian HE institutions showed the highest prevalence of job insecurity which corresponded with the highest level of perceived burnout in their work settings.

COVID-19 affected Australian HE institutions substantially because of their diversity, complexity, financial challenges, and international dependence. With closures of international borders, universities faced decreased student returns in 2020, and reduced numbers in 2021 and 2022, resulting in substantial revenue loss and 17,300 job losses on campuses in 2020 [23]. This reduction in job security appears to be associated with increased burnout in the Australian HE sectors.

Regarding coping, our study found that Australia had the lowest prevalence of medium to high resilient coping, consistent with the recent global study showing similarly low resilience amongst community members [9]. This may be attributed to increased top-down management and loss of control leading to tight deadlines, elevated workloads, contributing to higher burnout, and a high prevalence of mental health issues, compounded by living in a region experiencing the world’s longest lockdown [56, 57]. Australian tertiary students experienced low wellbeing and resilience, increasing the risk of future mental illness [58]. However, a previous study showed higher resilience prevalence (57%) among Australian residents [16]. In our study, Mexican staff exhibited the highest medium to high resilient coping levels aligning with a previous study showing that over 60% of Mexican university staff reported medium to high resilience coping [59], along with strong community support and cultural norms. Another recent study among Mexican faculty members found a strong relationship between burnout and resilience, demonstrating that adaptive mechanisms were acquired over time [60].

### Limitations

This multi-country study had limitations including uneven country representations due to some institutions having summer holidays during data collection. Mexico, the United Kingdom, and Australia contributed to 13–18% of the total participants, while other countries contributed 2–8%, potentially limiting country-wise analyses. Additionally, the study relied on self-reported data, potentially introducing recall bias or social desirability effects. Given the cross-sectional design, caution should be exercised when interpreting potential causal relationships between outcomes and other variables, as this represents an inherent limitation of the study design.

Differences in ethnicity, cultural backgrounds, public health policies, compliance with public health measures, and post-pandemic recovery stages across the 16 countries could have influenced the key issues investigated in this study. However, some of these variables were controlled during multivariate analysis. Despite these limitations, this global study was among the first to examine the health and wellbeing of staff at HE institutions worldwide. Collaboration between researchers ensured a substantial sample size with adequate power to enable robust examination of the aims and to provide valuable insights for developing psychological support strategies and interventions in HE institutions.

## Conclusions

This study identified several key health and wellbeing issues experienced by the staff working at HE institutions across 16 countries in the post-pandemic era. Although job insecurity was not pronounced, many staff reported perceived burnout, experienced moderate to high levels of psychological distress, and reported low adaptive coping. These vulnerable staff need support and services, and awareness of the existing resources. Based on the findings, it may be necessary to implement targeted policies or practices to address psychological distress and burnout issues more effectively. HE institutions could promote resilience-building initiatives to enhance staff health and wellbeing. The study found no single staff group vulnerable to all four key issues which were often interrelated. Thus, tailored efforts within robust mental health support structures are essential, along with a strong emphasis on the importance of fostering organisation support.

### Supplementary Information

Supplementary Material 1.

## Data Availability

All data generated or analysed during this study are included in this published article.
