# Supplementary material for: Health and wellbeing of staff working at higher education institutions globally during the post-COVID-19 pandemic period: evidence from a cross-sectional study
Source: BMC Public Health. 2024 Jul 11;24:1848. doi: 10.1186/s12889-024-19365-1 (PMC11238394; doi:10.1186/s12889-024-19365-1)
Supplement: Supplementary file 1 — Supplementary Material 1. [file 12889_2024_19365_MOESM1_ESM.docx]

**SUPPLEMENTARY**

**Table S.1: Response rate across 16 countries**

| No | Country | Organisation | Total staff (received emails) | Study participants | Response rate (%) |
| --- | --- | --- | --- | --- | --- |
| 1 | Australia | Federation University Australia | 2101 | 218 | 10 |
|  |  | Australian Catholic University | 2147 | 206 | 10 |
| 2 | China | Shenzhen University | 1200 | 137 | 11 |
| 3 | Hong Kong, China | The Chinese University of Hong Kong | 700 | 186 | 27 |
| 4 | Indonesia | Universitas Airlangga | 585 | 61 | 10 |
| 5 | Kuwait | Kuwait University | 397 | 96 | 24 |
| 6 | Malaysia | Universiti Malaysia Terengganu | 220 | 155 | 70 |
| 7 | Mexico | Universidad Anahuac Queretaro | 1158 | 316 | 27 |
| 8 | Oman | Oman College of Health Sciences-South Sharquiya | 500 | 86 | 6 |
|  |  | Sultan Qaboos University | 957 |  |  |
| 9 | Pakistan | International Islamic University | 870 | 37 | 4 |
| 10 | Palestine | Al Azhar University-Gaza | 665 | 91 | 14 |
| 11 | Saudi Arabia | Qassim University | 6900 | 47 | 1 |
| 12 | South Africa | University of Johannesburg | 1200 | 65 | 5 |
| 13 | Taiwan | Asia University | 364 | 46 | 13 |
| 14 | Turkey | Kirklareli University | 1133 | 147 | 13 |
| 15 | United Arab Emirates | University of Sharjah | 770 | 115 | 15 |
| 16 | United Kingdom | Northumbria University | 730 | 344 | 47 |
| TOTAL | | | 22597 | 2353 | 10 |

***Table S.2: Profession-related outcomes among the study participants***

| Characteristics | Total, n(%) |
| --- | --- |
| Total study participants | 2353 |
| Job Insecurity Scale (JIS) items | 2353 |
| Chances are, I will soon lose my job |  |
| Strongly disagree | 1059 (45.0) |
| Disagree | 717 (30.5) |
| Neutral | 399 (17.0) |
| Agree | 133 (5.7) |
| Strongly agree | 45 (1.9) |
| I am sure I can keep my job |  |
| Strongly disagree | 104 (4.4) |
| Disagree | 163 (6.9) |
| Neutral | 415 (17.6) |
| Agree | 824 (35.0) |
| Strongly agree | 847 (36.0) |
| I feel insecure about the future of my job |  |
| Strongly disagree | 596 (25.3) |
| Disagree | 667 (28.3) |
| Neutral | 471 (20.0) |
| Agree | 458 (19.5) |
| Strongly agree | 161 (6.8) |
| I think I might lose my job in the near future |  |
| Strongly disagree | 834 (35.4) |
| Disagree | 808 (34.3) |
| Neutral | 436 (18.5) |
| Agree | 191 (8.1) |
| Strongly agree | 84 (3.6) |
| JIS score (total) | 2353 |
| Mean (±SD) | 8.6 (±3.7) |
| Range | 4 to 20 |
| Job insecurity (JIS categories) | 2353 |
| No (total score 1-3) | 2004 (85.2) |
| Yes (total score 4-5) | 349 (14.8) |
| Perceived burnout | 2348 |
| Never | 268 (11.4) |
| A few times a year or less | 641 (27.3) |
| Once a month or less | 309 (13.2) |
| A few times a month | 446 (19.0) |
| Once a week | 169 (7.2) |
| A few times a week | 311 (13.2) |
| Every day | 204 (8.7) |
| Perceived burnout (Burnout scale categories) | 2348 |
| No (score 1-4) | 1664 (70.9) |
| Yes (score 5-7) | 684 (29.1) |

***Table S.3: Levels of psychological distress among the study participants***

| Characteristics | Total, n(%) |
| --- | --- |
| Total study participants | 2353 |
| Perceived status of own mental health | 2300 |
| Excellent | 325 (14.1) |
| Very good | 650 (28.3) |
| Good | 798 (34.7) |
| Fair | 414 (18.0) |
| Poor | 113 (4.9) |
| Perceived status of own mental health | 2300 |
| Poor to Fair | 527 (22.9) |
| Good to Excellent | 1773 (77.1) |
| K-10 items | 2304 |
| About how often did you feel tired out for no good reason? |  |
| None | 359 (15.6) |
| A little of the time | 615 (26.7) |
| Some of the time | 902 (39.1) |
| Most of the time | 352 (15.3) |
| All of the time | 76 (3.3) |
| About how often did you feel nervous? |  |
| None | 355 (15.4) |
| A little of the time | 768 (33.3) |
| Some of the time | 865 (37.5) |
| Most of the time | 270 (11.7) |
| All of the time | 46 (2.0) |
| About how often did you feel so nervous that nothing could calm you down? |  |
| None | 1212 (52.6) |
| A little of the time | 645 (28.0) |
| Some of the time | 367 (15.9) |
| Most of the time | 68 (3.0) |
| All of the time | 12 (0.5) |
| About how often did you feel hopeless? |  |
| None | 984 (42.7) |
| A little of the time | 667 (28.9) |
| Some of the time | 463 (20.1) |
| Most of the time | 167 (7.2) |
| All of the time | 23 (1.0) |
| About how often did you feel restless or fidgety? |  |
| None | 554 (24.0) |
| A little of the time | 840 (36.5) |
| Some of the time | 641 (27.8) |
| Most of the time | 228 (9.9) |
| All of the time | 41 (1.8) |
| About how often did you feel so restless you could not sit still? |  |
| None | 1193 (51.8) |
| A little of the time | 628 (27.3) |
| Some of the time | 380 (16.5) |
| Most of the time | 89 (3.9) |
| All of the time | 14 (0.6) |
| About how often did you feel so depressed? |  |
| None | 924 (40.1) |
| A little of the time | 755 (32.8) |
| Some of the time | 468 (20.3) |
| Most of the time | 127 (5.5) |
| All of the time | 30 (1.3) |
| About how often did you feel that everything was an effort? |  |
| None | 488 (21.2) |
| A little of the time | 709 (30.8) |
| Some of the time | 604 (26.2) |
| Most of the time | 408 (17.7) |
| All of the time | 95 (4.1) |
| About how often did you feel so sad that nothing could cheer you up? |  |
| None | 1059 (46.0) |
| A little of the time | 674 (29.3) |
| Some of the time | 437 (19.0) |
| Most of the time | 109 (4.7) |
| All of the time | 25 (1.1) |
| About how often did you feel worthless? |  |
| None | 1214 (52.7) |
| A little of the time | 578 (25.1) |
| Some of the time | 352 (15.3) |
| Most of the time | 120 (5.2) |
| All of the time | 40 (1.7) |
| K10 score (total) | 2304 |
| Mean (±SD) | 21 (7.4) |
| Range | 10 to 50 |
| Levels of psychological distress (K10 categories) | 2304 |
| Low (total score 10-15) | 619 (26.9) |
| Moderate (total score 16-21) | 716 (31.1) |
| High (total score 22-29) | 648 (28.1) |
| Very high (total score 30-50) | 321 (13.9) |
| Levels of psychological distress (K10 categories) | 2304 |
| Low (total score 10-15) | 619 (26.9) |
| Moderate to Very high (total score 16-50) | 1685 (73.1) |

***Table S.4: Coping during the post-pandemic period among the study participants***

| Characteristics | Total, n(%) |
| --- | --- |
| Total study participants | 2279 |
| BRCS items | 2279 |
| I look for creative ways to alter difficult situations |  |
| Does not describe me at all | 91 (4.0) |
| Does not describe me | 281 (12.3) |
| Neutral | 795 (34.9) |
| Describes me | 816 (35.8) |
| Describes me very well | 296 (13.0) |
| Regardless of what happens to me, I believe I can control my reaction to it |  |
| Does not describe me at all | 75 (3.3) |
| Does not describe me | 283 (12.4) |
| Neutral | 731 (32.1) |
| Describes me | 902 (39.6) |
| Describes me very well | 288 (12.6) |
| I believe I can grow in positive ways by dealing with difficult situations |  |
| Does not describe me at all | 47 (2.1) |
| Does not describe me | 218 (9.6) |
| Neutral | 623 (27.3) |
| Describes me | 1027 (45.1) |
| Describes me very well | 364 (16.0) |
| I actively look for ways to replace the losses I encounter in life |  |
| Does not describe me at all | 99 (4.3) |
| Does not describe me | 295 (12.9) |
| Neutral | 717 (31.5) |
| Describes me | 893 (39.2) |
| Describes me very well | 275 (12.1) |
| BRCS score (total) | 2279 |
| Mean (±SD) | 13.9 (3.3) |
| Range | 4 to 20 |
| Levels of coping (BRCS categories) | 2279 |
| Low resilient copers (score 4-13) | 962 (42.2) |
| Medium resilient copers (score 14-16) | 911 (40.0) |
| High resilient copers (score 17-20) | 406 (17.8) |
| Levels of coping (BRCS categories) | 2279 |
| Low resilient copers (score 4-13) | 962 (42.2) |
| Medium to High resilient copers (score 14-20) | 1317 (57.8) |
| Did anything differently to cope with stress | 2273 |
| No | 1153 (50.6) |
| Yes | 1120 (49.1) |
